# Evaluation of the Effects of a Monthly Buprenorphine Depot Subcutaneous Injection on QT Interval During Treatment for Opioid Use Disorder

**DOI:** 10.1002/cpt.1406

**Published:** 2019-04-08

**Authors:** Virginia D. Schmith, Laura Curd, Lauren R. L. Lohmer, Celine M. Laffont, Anne Andorn, Malcolm A. Young

**Affiliations:** ^1^ Nuventra Pharma Sciences Durham North Carolina USA; ^2^ Indivior Inc. Richmond Virginia USA

## Abstract

Extensive 12‐lead electrocardiogram monitoring and drug concentrations were obtained during development of BUP‐XR, a monthly subcutaneous injection for the treatment of opioid use disorder (OUD). Matched QT and plasma drug concentrations (11,925) from 1,114 subjects were pooled from 5 studies in OUD. A concentration‐QT model was developed, which accounted for confounding factors (e.g., comedications) affecting heart rate and heart rate‐corrected QT interval (QTc). Bias‐corrected nonparametric two‐sided 90% confidence intervals (CIs) were derived for the mean predicted effect of BUP‐XR on QTc (ΔQTc) at therapeutic and supratherapeutic doses. Changes in QTc were associated with age, central vs. noncentral reading, sex, methadone, and barbiturates. The upper 90% CI of ΔQTc was 0.29, 0.67, and 1.34 ms at the steady‐state peak concentration (C_max_) for 100, 300, and 2 × 300 mg doses, respectively. An effect of BUP‐XR on QT can be ruled out at therapeutic and supratherapeutic doses of BUP‐XR, after accounting for covariates that may influence heart rate and QT interval in OUD.

**Study Highlights**

**WHAT IS THE CURRENT KNOWLEDGE ON THE TOPIC?**

☑There are conflicting results around whether buprenorphine can cause increases in QT interval. In addition, QT interval changes are difficult to assess in subjects with opioid use disorder (OUD). There is no true baseline given the large number of concomitant medications used to treat withdrawal symptoms and illicit drugs that can cause changes in QT interval or heart rate.

**WHAT QUESTION DID THIS STUDY ADDRESS?**

☑Does buprenorphine increase QTc at therapeutic or supratherapeutic BUP‐XR doses, after accounting for other drugs that may affect the QTc interval?

**WHAT DOES THIS STUDY ADD TO OUR KNOWLEDGE?**

☑The effect of buprenorphine on QTc could be ruled out at therapeutic and supratherapeutic concentrations after administration of BUP‐XR.

**HOW MIGHT THIS CHANGE CLINICAL PHARMACOLOGY OR TRANSLATIONAL SCIENCE?**

☑A novel method for evaluating the concentration‐QT relationship in the presence of concomitant medications that affect heart rate or QT interval is presented.

Buprenorphine formulated in the well‐established ATRIGEL Delivery System (BUP‐XR, AMRI, Burlington, MA) is the first s.c. injected, extended‐release, monthly buprenorphine formulation approved by the US Food and Drug Administration (FDA) for use in patients with moderate to severe opioid use disorder (OUD). Buprenorphine has a well‐established safety profile. Several studies have reported that buprenorphine does not have a substantial effect on QT,^1^, ^2^, ^3^ with average concentrations of 1.6–2 ng/mL.^3^ Buprenorphine and naloxone did not prolong the QT interval when administered alone, but did increase QT when administered with delavirdine or ritonavir.^4^ In contrast, a thorough QT study of transdermal buprenorphine failed to exclude a 10‐ms increase in QT at the highest transdermal dose studied (40 mg).^5^ Sublingual buprenorphine administered with naltrexone to healthy volunteers had no effect on heart rate‐corrected QT (QTc) at buprenorphine plasma levels ≤ 5 ng/mL.^6^ However, although designed to eliminate the confounding effects of changes in heart rate (HR) on QT with the use of naltrexone, differences in HR (≤ 5 bpm) were still observed. Thus, the effect of buprenorphine on QTc remains unclear.

The pivotal phase III efficacy and safety trial of BUP‐XR (RB‐US‐13‐0001) was a double‐blind, randomized, placebo‐controlled study evaluating the two approved dosing regimens for BUP‐XR. Both regimens started with two consecutive monthly doses of 300 mg, followed by monthly maintenance doses of 100 mg (300/100 mg regimen) or 300 mg (300/300 mg regimen). At steady state, average plasma buprenorphine concentrations were 3.21 ng/mL and 6.54 ng/mL for the 300/100 mg and 300/300 mg regimens, respectively.^7^ As expected, BUP‐XR s.c. injection resulted in less fluctuation in plasma buprenorphine concentrations than after sublingual dosing, with lower peak concentrations (C_max_) for similar level of exposure and a median time of maximum concentration (T_max_) of ~ 24 hours.^8^ This study included collection of electrocardiograms (ECGs; through Holter monitoring for 24 hours after each injection), serial 12‐lead ECGs, and serial blood samples (for assessment of plasma concentrations of buprenorphine and its metabolite, norbuprenorphine) at specific times throughout the dosing interval.

The concentration‐QT model was developed using data from five BUP‐XR clinical studies: four phase I studies and one phase III study (**Table** 1), where subjects may have used illicit drugs and could receive comedications to alleviate their withdrawal symptoms. Some of these comedications and illicit drugs have the potential to affect QT or HR (**Figure** 1). In addition, in most studies, subjects may have received sublingual buprenorphine for induction prior to receiving BUP‐XR. Therefore, there was no true baseline QT interval or HR in subjects with OUD (even when on placebo). The goal of the present analysis was to account for the effect of withdrawal, comedications, and illicit opioids on HR or QT, utilizing subjects' urinary drug screens and self reports, prior to establishing whether there is a drug‐related effect of buprenorphine on QT.

**Table 1:** **Summary of studies included in analysis**

Study design	Dose and dosing regimen	Blood sample and 12‐lead ECG collection (when collected at the same time)
Phase III pivotal efficacy and safety study		
A randomized, double‐blind, placebo‐controlled, multicenter study to assess the efficacy, safety, and tolerability of multiple s.c. injections of BUP‐XR (100 and 300 mg) over 24 weeks in treatment‐seeking subjects with OUD (RB‐US‐13‐0001) 504 subjects were randomized in a 4:4:1:1 ratio to BUP‐XR 300/100 mg, BUP‐XR 300/300 mg, placebo matched to 300/100 mg, placebo matched to 300/300 mg	Open‐label run‐in with sublingual buprenorphine/naloxone film (3‐day induction and 4–11‐day dose adjustment) to achieve buprenorphine doses ranging from 8 to 24 mg/day. Then, on day 1, subjects were randomized to the following treatments with doses given every 28 (±2) days: 300/300 mg: BUP‐XR 300 mg × 6 injections 300/100 mg: BUP‐XR 300 mg × 2 injections followed by 100 mg × 4 injections Placebo: volume‐matched injections × 6^b^	Matched blood samples and single 12‐lead ECG measurements were taken on days −1, 8, 15, 22, 36, 43, 50, 64, 71, 78, 92, 99, 106, 120, 127, 134, 148, 155, 162, 169, and 197; Holter ECG measurements were available on dosing days from predose through 24 hours (days 1, 2, 29, 30, 57, 58, 85, 86, 113, 114, 141, and 142) and specific 12‐lead ECG tracings of 10‐second duration were extracted in triplicate prior to injection and 4 and 24 hours postdose. PK samples were taken at 1 and 4 hours postdose on dosing days Holter data on study dosing days were manually read and included triplicate readings. All other ECGs were non‐Holter readings in triplicate (at screening) or as a single reading (for all others). ECG measured at screening.
Phase I studies		
A single‐dose, open‐label study of BUP‐XR in opioid‐dependent individuals (RB‐US‐10‐0011) 12 subjects received BUP‐XR	BUP‐XR 20 mg single dose	Matched blood samples and single 12‐lead ECG measurements were collected on day 1 at 3 hours^a^ and 12 hours postdose and days 2, 3, 8, 14, and 25–30 (prior to methadone and/or 4 hours post‐methadone dose), 32, 57, 85, and 120. ECG measured at screening, day −2 and day 1 predose.
An open‐label, single ascending‐dose study to evaluate the safety, tolerability, and PK of BUP‐XR in opioid‐dependent subjects (RB‐US‐11‐0020) 51 subjects received BUP‐XR	Cohorts 1–3: Single dose of BUP‐XR 50 mg, 100 mg, or 200 mg Cohort 4: Single dose of BUP‐XR 100 mg, following a 7‐day run‐in with sublingual buprenorphine/naloxone tablets to achieve a stable buprenorphine dose of 12 mg/day	Matched blood samples and single 12‐lead ECG measurements were collected on days 1 at 3 hours^c^ and 12 hours), 2, 3, 7, 14, 16–21, 35, 42, 63, 84, 112, and 150 and if subjects received methadone, (4 hours post‐methadone dose each day. ECG measured on days −9 (cohort 4 only), −2, −1 and day 1 predose^d^ (from cohorts 1−3 only) and screening (from all cohorts).
An open‐label, multiple dose study of the safety, tolerability, PK, efficacy markers, and opioid receptor availability of s.c. injections of depot BUP‐XR in treatment‐seeking opioid‐dependent subjects (RB‐US‐12‐0005) 89 subjects received BUP‐XR	Repeated doses (≥ 4 injections) of BUP‐XR 50, 100, 200, and 300 mg every 28 days, following a 13‐day run‐in with sublingual buprenorphine tablets to reach stable buprenorphine doses of 8, 12, 14, 24 mg or 8–24 mg	Matched blood samples and 12‐lead ECG measurements were taken on day −5 (run‐in), 1 (at predose^d^ and 12 hours), 3, 29 (2 and 12 hours), 31, 57 (predose and 12 hours), 59, 85 (predose and 12 hours), 87, and 141. Subjects receiving 300 mg BUP‐XR had more dosing days planned and additional matched PK and ECG measurements on days 113 (predose and 12 hours), 115, 141 (predose and 12 hours), 143, and 197. ECG measured at screening.
A single‐center, randomized, open‐label, single‐dose study to evaluate the PK, safety, and tolerability of BUP‐XR using poly (DL‐lactide‐co‐glycolide) polymer of two different molecular weights (low and high molecular weights as test treatments) in comparison to intermediate molecular weight (reference treatment) in treatment‐seeking subjects with OUD (RB‐US‐13‐0006) 47 subjects were randomized to receive BUP‐XR	Single dose of BUP‐XR 300 mg, following 7−8‐day run‐in with sublingual buprenorphine/naloxone film to achieve a stable buprenorphine dose of 12 mg/day	Matched blood samples and 12‐lead ECG measurements were taken on day 1 (predose^d^ and 12 hours), day 2 (24 and 36 hours), day 3 (48 hours), days 4, 6, 14, 27, and 57. ECG measured at screening.

ECG, electrocardiogram; OUD, opioid use disorder; PK, pharmacokinetic(s); SL, Sublingual.

^a^Additional blood samples or ECG measurements may be collected, but only simultaneous measurements were used in the analysis. ^b^Placebo data cannot be used before day 14 because of the SL buprenorphine/naloxone run‐in and lack of PK samples; values > day 14 were assumed to have concentrations of buprenorphine and norbuprenorphine equal to 0. ^c^Three‐hour ECG data matched with either the 2‐hour or 4‐hour blood sample, depending on which was closer in time to the actual collection of the ECG. ^d^Predose ECG and buprenorphine data on day 1 can only be matched if there is a quantifiable buprenorphine concentration.

**Figure 1:**
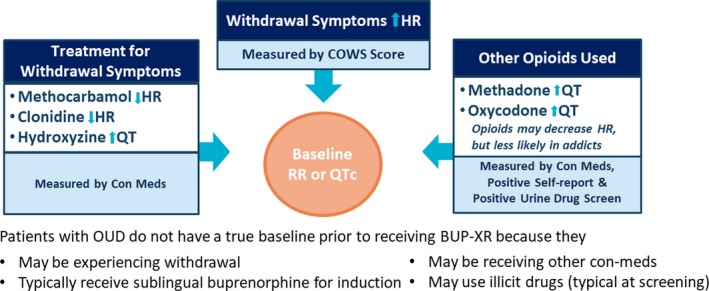
Challenges in identifying baseline heart rate (HR) and QT interval and evaluating any effects of buprenorphine on HR and QT interval. COWS, Clinical Opioid Withdrawal Scale; OUD, opioid use disorder; QTc, heart rate‐corrected QT interval; RR, RR interval.

Given the large number of comedications that may affect HR, the standard Fredericia correction (QTcF; where alpha = 0.333) or individual correction (QTcI; where alpha is estimated in individual subjects) may not be appropriate. As long as a drug does not affect HR, QTcF and QTcI are likely to produce similar results.^6^ However, if there is an effect of a drug on HR of ≥ 5 bpm, QTcF and QTcI can lead to conflicting conclusions of whether there is an effect on QT.^6^ Although there is not a consensus on the best method for evaluating the QT risk of drugs that affect HR, Garnett *et al*.^6^ suggests the use of a different alpha on and off drug to correct for differences in the QT‐RR relationship. This approach was applied in the present analysis.

The rationale for the present analysis was to characterize the concentration‐related effect of buprenorphine or norbuprenorphine on QT interval in OUD subjects but only after accounting for the effects of withdrawal, relevant comedications, and illicit drug use on HR or QT. A model was developed so that the concentration‐related effects of buprenorphine on QTc interval could be predicted at therapeutic and supratherapeutic concentrations of BUP‐XR.^9^ Results from this concentration‐QT approach were used with summarization of the average and individual observed and change from baseline QTc and HR data^10^ to inform the proarrhythmic risks of BUP‐XR.

## Results

The full dataset included 11,925 concentration‐QT observations from 1,114 subjects, which was used in the evaluation of the effects of buprenorphine and covariates on QTc. A reduced dataset, which only included data from baseline (in the absence of buprenorphine) or placebo (after discontinuation of sublingual buprenorphine/naloxone) included 2,210 observations from 1,099 subjects and was used in the evaluation of covariates on HR.

### Demographics and patient covariates

The median age was 36 years (range 19–65 years with 70% men. The average Clinical Opioid Withdrawal Scale (COWS) scores were mild (range 2–7) for each study prior to receiving BUP‐XR. Approximately 40% of ECGs collected in study RB‐US‐13‐0001 were from Holter monitoring, whereas all remaining records had non‐Holter readings. Triplicates and central readings were only done for study RB‐US‐13‐0001.

Studies, patient populations, and dosing schedules are described in **Table** 1. Comedications are summarized in **Table** S1.

### Exploratory analysis

**Figure 2:**
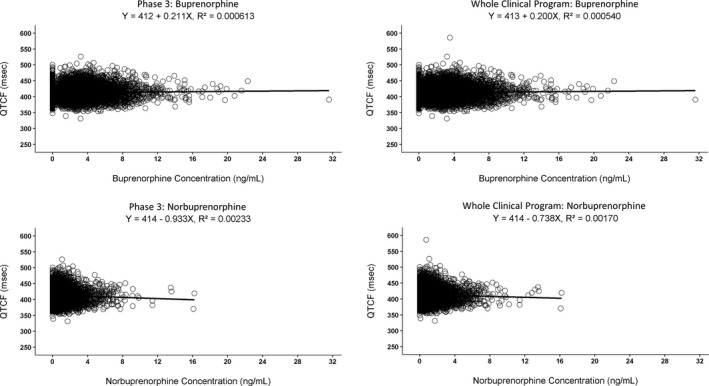
Time‐matched concentration‐QTc for the phase III program and the whole clinical program. Circles represent individual observations; black solid line represents the results from linear regression. The equation with the *r* ^2^ is also presented. QTcF, QT after Fredericia correction.

There was no counter‐clockwise hysteresis based on phase I studies. There was no apparent relationship between plasma buprenorphine concentrations and QTcF (**Figure** 2) based on observations. Exploratory plots indicated no effects of covariates on RR interval that were > 200 ms or on QTcF interval that were ≥10 ms.

### Model development

The base model for QTc using the reduced dataset included a parameter describing the QTc in the absence of buprenorphine (QTc_Abs_) of 407 ms in men and 414 ms in women with interindividual variability (IIV) of ± 15 ms. Fixing alpha to 0.333 with no IIV was chosen given there was no improvement in the description of the QT‐RR relationship with QTcI and the η shrinkage was high (43%).

The only covariate found to influence alpha was the COWS score, where alpha ranged from 0.353−0.415 for COWS score between 5 (mild) and 20 (moderate).

Backward elimination of each covariate on QTc_Abs_ using the full dataset resulted in the following statistically significantly effects on QTc_Abs_: age (+17‐ms increase for 70 years compared with 18 years), methadone (+6.5 ms), Holter vs. non‐Holter (−1.7 ms), central vs. noncentral reading (−7.4 ms), and hydroxyzine (+1.8 ms).

There were no concentration‐related effects of buprenorphine or norbuprenorphine on RR interval. The concentration‐related slope on QTc_Abs_ for buprenorphine and norbuprenorphine were both close to zero (but negative), with norbuprenorphine having an even steeper negative slope. The buprenorphine concentration‐related slope on QTc_Abs_ was chosen as conservative because the slope was less negative with a much larger SE (i.e., having the highest likelihood of an upper 90% confidence interval (CI) that is positive).

Diurnal variation was tested but not retained because the amplitude was < 1 ms and the concentration‐related slope did not change substantially (−0.06 ms/ng/mL vs. 0.0567 ms/ng/mL). The model was refined by evaluating any potential bias in conditional weighted residuals (CWRES) vs. covariates.

Final covariates selected included: age (+16.8‐ms increase for 70 years compared with 18 years) on QTc_Abs_, central vs. noncentral reading (−8.4 ms) on QTc_Abs_, sex (+7.6 ms in women) on QTc_Abs_, methadone (+6.1 ms) on QTc_Abs_, barbiturates (+5 ms) on QTc_Abs_, phencyclidine (+3.4 ms) on QTc_Abs_, hydroxyzine (+1.7 ms) on QTc_Abs_, cocaine (+1.7 ms) on QTc_Abs_, Holter vs. non‐Holter (−1.7 ms) on QTc_Abs_, oxycodone (−1.5 ms) on QTc_Abs_, codeine (+1.3 ms) on QTc_Abs_, and an effect of COWS on alpha (alpha = 0.341–0.363 for a COWS score between 5 and 20, respectively). Important parameters (e.g., concentration‐related slope, relative uncertainty, and magnitude of each covariate effect) did not differ significantly between models before and after model refinement.

### Final model

Final model parameters are given in **Table** 2. All parameters were estimated with adequate precision except the buprenorphine concentration‐related slope (as expected given the lack of a strong relationship). The concentration‐related slope was negative, with a −1.6‐ms difference at the highest observed concentration (32 ng/mL).

**Table 2:** **Summary of estimates from final model using NONMEM and a nonparametric bootstrap**

	NONMEM		Bootstrap			
	Estimate	% RSE^a^	Median	% RSE^a^	2.5th	97.5th
QTc_Abs_ (ms)	400	0.2	400	0.4	396	403
Alpha	0.333	Fixed	0.333	Fixed	—	—
Sex on QTc_Abs_ b	0.0189	13.7	0.01911	13.8	0.0140	0.0241
COWS on alpha (slope)	0.00151	52.1	0.00151	51.5	−0.0000274	0.00307
Hydroxyzine on QTc_Abs_ b	0.00423	35.7	0.00422	36.3	0.00115	0.00715
Methadone on QTc_Abs_ b	0.0153	18.4	0.01522	17.4	0.0101	0.0210
Age on QTc_Abs_ (ms/year)	0.324	7.0	0.32158	12.9	0.232	0.406
Holter vs. computerized on QTc_Abs_ b	−0.00423	24.6	−0.00421	25.2	−0.00624	−0.00206
Central vs. noncentral reading on QTc_Abs_ b	−0.021	11.5	−0.02097	13.1	−0.0266	−0.0158
Concentration‐related slope (ms/ng/mL)^b^	−0.0507	168.8	−0.04617	181.1	−0.225	0.110
Codeine on QTc_Abs_ b	0.00327	35.2	0.00333	33.7	0.00102	0.00551
Oxycodone on QTc_Abs_ b	−0.00378	43.4	−0.00381	42.9	−0.00694	−0.000448
Phencyclidine on QTc_Abs_ b	0.00861	44.1	0.00902	42.3	0.000451	0.0159
Barbiturates on QTc_Abs_ b	0.0124	38.9	0.01223	40.4	0.00205	0.0219
Cocaine on QTc_Abs_ b	0.00428	28.0	0.00427	28.0	0.00193	0.00651
IIV on QTc_Abs_ (ms)	14.3	5.2	14.2	5.2	13.5	15
IIV on concentration‐related slope (ms/ng/mL)	0.767	33.1	0.748	34.8	0.5	1
RE (ms)	10.6	3.3	10.6	3.1	10.3	11

COWS, Clinical Opioid Withdrawal Scale; IIV, interindividual variability; QTc, heart rate‐corrected QT; QTc_Abs_, base model for QTc using the reduced dataset included a parameter describing the QTc in the absence of buprenorphine.

^a^RSE = relative standard error (100 × SE divided by the estimate). ^b^Fractional change in QTc_Abs_.

Primary goodness‐of‐fit plots are given in **Figure** S1. There was no bias in population predictions (PRED) vs. observations (DV), individual predictions vs. dependent variable (DV), or CWRES vs. time plots. Plasma norbuprenorphine concentration vs. CWRES showed no bias. Thus, there was no evidence of any additional effect of metabolite on QTc.

### Model performance

Results from the nonparametric bootstrap are included in **Table** 2. Parameter estimates were nearly identical to those estimated by NONMEM. Visual predictive checks (VPCs) of data from study RB‐US‐13‐0001 showed that the model appropriately captured the buprenorphine concentration‐QT relationship and the QT‐time profile (**Figures S2 and S3**).

### Sensitivity analysis

Three sensitivity analyses were conducted to evaluate the influence on the concentration‐related slope:

- QTcI instead of QTcF
- Data from study RB‐US‐13‐0001 alone (given differences in collection of ECGs)
- Elimination of the age‐related slope on QTc_Abs_

The results from these sensitivity analyses are given in **Table** S2. Using QTcI instead of QTcF resulted in a slightly more negative slope for buprenorphine concentration (−0.0604 compared to −0.0507, respectively). Phase III data alone resulted in a less negative slope than for all data (−0.0157 compared to −0.0507, respectively). Eliminating age from the model resulted in an increase in the objective function value (OFV; *P* < 0.0001), did not substantially change the concentration‐related slope (−0.0431 compared to −0.0507), but significantly increased the baseline QTc_Abs_ in men to 411 ms (compared with 400 ms). Final modeling results were consistent with plots of QTcF interval vs. age (**Figure** S4), where the age‐related slopes were 0.357 ms/year and 0.202 ms/year for the reduced and full datasets, respectively, compared with the model‐based slope of 0.324 ms/year.

### Predicted concentration‐related changes in QTcF at therapeutic and supratherapeutic concentrations

**Figure 3:**
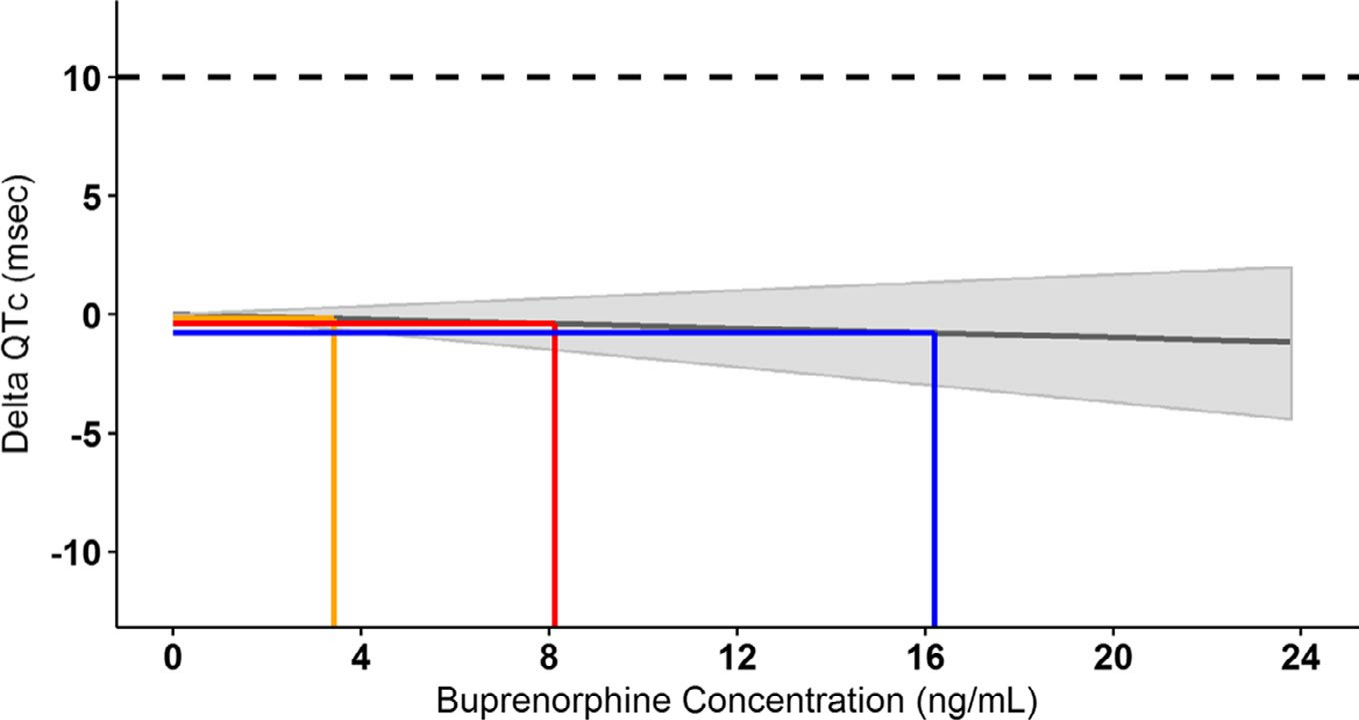
Predicted mean delta heart rate‐corrected QT (QTc) at various buprenorphine concentrations with the 90% confidence interval (shaded), including steady‐state 100‐mg (orange) and 300‐mg (red) concentrations and supratherapeutic concentrations (blue). Solid line represents the predicted delta QTc at various buprenorphine concentrations with the shaded area representing the 90% confidence interval; the orange line represents the geometric mean peak concentration (C_max_) at 100 mg every 28 days; the red line represents the geometric mean C_max_ at 300 mg every 28 days, and the blue line represents the geometric mean C_max_ at 2 × 300 mg every 28 days.

The predicted mean delta QTc (ΔQTc) by buprenorphine concentration is illustrated in **Figure** 3 and presented in **Table** 3. The upper 90% CI is under 10 ms for maintenance doses of 100 and 300 mg and supratherapeutic concentrations of 2 × 300 mg doses, ruling out an effect of BUP‐XR on QTc.

**Table 3:** **Mean, median, and 90% CIs for the geometric mean C**_**max**_ **and the delta QTc and the bias‐corrected 90% CI of the upper bound**

Maintenance dose (mg)	Geometric mean C_max_ (ng/mL)			Delta QTc (ms)			
	Mean	Median	90% CI	Mean	Median	90% CI	Bias‐corrected 90% CI
100^a^	3.44	3.43	3.25–3.63	−0.17	−0.16	−0.65 to 0.29	−0.65 to 0.29
300^b^	8.12	8.12	7.54–8.72	−0.40	−0.38	−1.52 to 0.66	−1.52 to 0.67
2 × 300^c^	16.2	16.2	15.1–17.4	−0.79	−0.75	−3.04 to 1.32	−3.05 to 1.34

CIs, confidence intervals; C_max_, peak concentration; QTc, heart rate‐corrected QT.

^a^The geometric mean C_max_ for BUP‐XR 100 mg at steady‐state from study RB‐US‐12‐0005 (cohorts 2 and 4, injection 4) and study RB‐US‐13‐0001 (300/100 mg treatment group receiving 300 mg × 2 followed by 100 mg × 4, injection 6). ^b^The geometric mean C_max_ for BUP‐XR 300 mg at steady state were calculated using 300 mg data from study RB‐US‐12‐0005 (cohort 6, injection ≥ 4) and study RB‐US‐13‐0001 (300/300 mg treatment group receiving 300 mg × 6, injection 6). ^c^The geometric mean C_max_ for supratherapeutic concentrations was obtained as the geometric mean C_max_ for 300 mg (the highest dose) multiplied by a factor of 2 (not studied).

## Discussion

A concentration‐QT model developed from concentration‐ECG data across five studies with rigorous ECG monitoring has ruled out an effect of buprenorphine on QT at therapeutic and supratherapeutic plasma buprenorphine concentrations following BUP‐XR administration, after accounting for the covariates that may influence HR and QTc in subjects with OUD. The analysis followed the relevant International Conference on Harmonization guidelines,^9^ with a prospective analysis plan that outlined the modeling methods and assumptions, criteria for model selection, rationale for model components, and potential for pooling of data across studies. Importantly, the analysis utilized robust, high‐quality ECG data with almost 12,000 observations from 1,114 subjects and included sensitivity analyses to evaluate the effect of various assumptions.

This finding is consistent with the overall safety data from study RB‐US‐13‐0001 where only seven patients had an increase from baseline QTc > 60 ms (2/203 patients (1.0%) in the 300/100 mg group and 5/201 patients (2.0%) in the 300/300 mg group) and one patient in the 300/300 mg group was found to have a QTc > 500 ms. These findings were all sporadic and transient and none led to aberrant ventricular rhythm. Review of ECG and adverse event data provided no evidence for syncope, seizure, or ventricular tachycardia or fibrillation.

### Covariate effects

Withdrawal from opioids and drugs used to treat these symptoms (e.g., clonidine and methocarbamol) can increase the HR, which can influence QTc interval given the well‐known QT‐RR relationship. Only withdrawal symptoms had a significant effect on alpha, which was very small, with an alpha of 0.341 for a COWS score of 5 (mild) and 0.363 for a COWS score of 20 (moderate).

Although drugs used to treat withdrawal symptoms can increase QT, the present analysis showed that the effect of hydroxyzine on QTc was very small. An effect of methadone was observed as expected,^11^ because two phase I studies had data with and without methadone administration in the same subjects. The magnitude of change due to methadone was lower than expected, likely because data were combined across methadone doses (20–100 mg) and times (e.g., all buprenorphine times plus methadone's T_max_). The effect of barbiturates and codeine on QT were consistent with those reported previously^11^, ^12^ as was the small effect of cocaine on QT.^11^, ^13^ The effect of phencyclidine observed on QT has not been reported, but is likely related to changes in HR.^14^ The oxycodone effect was small and opposite of expectations.^12^ Importantly, the goals of including comedications were to reduce the signal‐to‐noise ratio by accounting for all factors affecting QT prior to characterizing the effect of buprenorphine on QT. The 90% CIs for ΔQTc were very narrow showing that these goals were met.

The effect of sex on QTc was anticipated; however, the effect of age (+16.8‐ms increase in a 70‐year‐old compared with an 18‐year‐old subject) on QTc was larger than expected. This age effect was consistent with observed data (**Figure** S4), where the age‐related slopes were similar to the model‐based slope. The magnitude of this effect was confirmed in the sensitivity analysis where QTc_Abs_ increased substantially, but the concentration‐related slope did not change when age was eliminated from the model. Although the reason for this age effect is unknown, it may be related to long‐term consequences of intake of comedications or illicit drugs by OUD subjects.

The changes in QTc related to central vs. noncentral reading were large; central reading was only performed in study RB‐US‐13‐0001, so this may be a confounding factor. A small effect due to Holter vs. non‐Holter readings was found, which is based on the large number of measurements of both in study RB‐US‐13‐0001. Given that a sensitivity analysis using data from study RB‐US‐13‐0001 alone resulted in a slightly smaller concentration‐related slope, this effect of Holter vs. non‐Holter and central vs. noncentral readings did not affect the conclusions from the study.

### Comparison with published information

The lack of an effect of buprenorphine on QTc in the present analysis is consistent with some reports of buprenorphine^1^, ^2^, ^3^ but not with others, including results from a healthy volunteer study^15^ and a buprenorphine transdermal system study.^5^ The discrepancy may be due to differences between subject populations, where healthy volunteers are more likely to have larger changes in HR than OUD subjects^16^ or the confounding effects of changes in HR in healthy volunteers could not be completely eliminated by the addition of naltrexone.^15^ Importantly, almost 12,000 matched buprenorphine concentration and robust high‐quality ECGs were analyzed from the population of interest (OUD), ruling out an effect of buprenorphine on QT at therapeutic and supratherapeutic concentrations.

## Materials and Methods

Matching plasma buprenorphine/norbuprenorphine concentrations and 12‐lead ECGs were pooled from the four phase I clinical studies and the phase III pivotal efficacy study conducted with BUP‐XR in OUD subjects (**Table** 1). The large phase III study had single or triplicate 12‐lead ECG measurements collected with and without Holter monitoring from 866 subjects (9,264 samples) included in the analysis, where 437 subjects have matched screening records but were not randomized, and 429 subjects were randomized to receive the following treatments with doses given every 28 (±2) days:

- Three hundred/100 mg: BUP‐XR 300 mg × 2 injections followed 100 mg BUP‐XR × 4 injections
- Three hundred/300 mg: BUP‐XR 300 mg × 6 injections
- Placebo: volume‐matched to BUP‐XR 300/100 mg group or 300/300 mg group

All studies were conducted in accordance with the ethical standards of the responsible committee on human experimentation or with the Helsinki Declaration of 1975 (as revised in 1983). Institutional review board approval was obtained prior to enrollment of subjects.

Most studies included an induction/dose stabilization period using sublingual buprenorphine prior to BUP‐XR dosing. As a result, there were no true baseline measurements in most studies prior to BUP‐XR administration. In study RB‐US‐13‐0001, subjects received sublingual buprenorphine/naloxone before randomization to BUP‐XR or placebo on day 1 and for up to 5 days after randomization. Because related plasma buprenorphine concentrations were not analyzed for placebo subjects, only data greater than or equal to day 14 from subjects randomized to placebo were included in the analysis.

### Blood sampling and 12‐lead ECG collection

Blood samples (for determination of plasma buprenorphine and norbuprenorphine concentrations) and 12‐lead ECGs were collected as outlined in **Table** 1. In study RB‐US‐13‐0001, triplicate ECGs were collected at screening, single 12‐lead ECGs were collected at relevant times on nondosing days, and triplicate readings were collected from Holter monitoring on dosing days at 4 and 24 hours postdose. Only Holter and non‐Holter data from study RB‐US‐13‐0001 were centrally read; non‐Holter ECG data from all other studies were reviewed at the site for any abnormalities.

### Concentration‐QT dataset

Only paired concentration‐QT measurements (e.g., with difference < 1 hour up to 24 hours after dosing, < 2 hours between 24 and 96 hours after dosing; or on the same day thereafter for BUP‐XR) were included in the analysis. Only plasma buprenorphine concentrations that were quantifiable, considered below the limit of quantification (set to 0), or if during screening with a negative urinary drug screens and self report for buprenorphine (set to 0) were included. In those subjects receiving placebo in study RB‐US‐13‐0001, plasma buprenorphine concentrations were set to 0 for samples collected ≥ 14 days after the first dose of placebo.

If a comedication has a well‐established effect on HR or QT and there were ≥ 25 subjects receiving this medication, then a time‐varying variable was added. Otherwise, the subject's data were excluded. In total, 98 subjects were excluded (7.1% of overall dataset) with 73 subjects who received drugs that may prolong QT, 2 subjects who received drugs known to increase or decrease HR, and 23 subjects from one site in study RB‐US‐13‐0001 closed by the sponsor following consultation with the FDA (secondary to protocol noncompliance).

### Concentration‐QT modeling

Population concentration‐QT modeling was performed using NONMEM version 7.3 (ICON Development Solutions, Ellicott City, MD). All graphical analyses were performed using R version 3.0.2 or later (R Foundation for Statistical Computing, Vienna, Austria). Goodness‐of‐fit plots were performed using the xpose4 package in R. Bootstrapping and VPCs were conducted using Perl‐speaks‐NONMEM program version 3.4.2.

The analysis plan stated that the reduced dataset (where plasma buprenorphine concentrations and norbuprenorphine concentrations were equal to 0) was to be used for development of a covariate model (prior to the incorporation of a buprenorphine concentration‐related slope), unless the number of important comedications was too small for evaluation, and that diurnal variation was only to be assessed after the buprenorphine (and/or norbuprenorphine) concentration‐related effects on QT had been added. Because plots showed different relationships between covariates (e.g., methamphetamines, hydroxyzine, and methadone) and RR or QT in the reduced vs. full datasets and because the reduced dataset was substantially smaller (2,210 observations) than the full dataset (11,925 observations), the decision was made to use the reduced dataset for the evaluation of covariate effects on HR (alpha) and then to switch to the use of the full dataset for evaluation of all other covariate effects on QTc.

### Base model of QT and RR intervals

The base model was fit to all QT and RR interval data using Eq. (1)

(1) $$\text{QT}\;=\;{\text{QTc}}_{\mathrm{Abs}}*{\left(\frac{\text{RR}}{1,000}\right)}^{\mathrm{alpha}}$$

where QTc_Abs_ was estimated as representing the QTc in the absence of buprenorphine and alpha was fixed to 0.333 (Frederica's correction). IIV was added to QTc_Abs_ using an additive error model. Residual (unexplained) error on QT was modeled using an additive error model.

### Addition of covariate effects to alpha or QTc_Abs_

The effects of gender on baseline (QTc_Abs_) were evaluated using Eq. (2)

(2) $${\text{QTc}}_{\mathrm{Abs}}=\text{THETA}\left(1\right)*\left(1+{\text{SLP}}_{\mathrm{sex}}*\text{SEX}\right)$$

where THETA(1) is the estimated QTc_Abs_ in men (SEX = 0) and SLP_sex_ is the fractional increase in QTc_Abs_ for women (SEX = 1).

Next, the following covariates were evaluated on alpha based on their potential to affect HR: withdrawal symptoms (as measured by COWS); clonidine and methocarbamol (used to treat withdrawal symptoms); cocaine, phencyclidine, cannabinoids, barbiturates, and methamphetamines (illicit drugs); amphetamines (taken for therapeutic (e.g., attention deficit hyperactivity disorder) or illicit use); and albuterol (known to increase HR). Then, the following covariates were evaluated on QTc: sex, age, opioids that have well‐established effects on QTc (methadone and oxycodone), opioids that may have an effect on QTc (hydrocodone, morphine, hydromorphone, oxymorphone, heroin, and codeine) or opioids (as a general class), benzodiazepines, barbiturates, triplicate vs. single ECG readings, central vs. non centrally read ECGs, and Holter vs. non‐Holter.

The analysis plan outlined that covariates were to be assessed using univariate analysis (change in OFV of at least 6.635 (*P* ≤ 0.01, with 1 degree of freedom)) followed by backward elimination of an increase in the OFV of at least 10.828 (*P* ≤ 0.001, with 1 degree of freedom). The decision to include a covariate was not to be based solely on the change in the OFV. In addition, theoretical rationale, goodness‐of‐fit plots, the precision of estimates, exploration of empirical Bayes estimates vs. covariates, and shrinkage were considered, with ultimate goal to describe a conservative concentration‐QT model with adequate precision for simulation. If ≥ 2 covariate effects were highly correlated, then the model with the largest slope was to be included in the model.

Each predefined covariate was evaluated univariately on alpha or on QTc_Abs_, with the exception of those that were correlated. Using this approach, methadone did not have an effect on QTc_Abs_, which raised the possibility that univariate analysis was not appropriate given that subjects received many drugs at one time (i.e., 49% with ≥ 3 drugs, 37% with ≥ 4 drugs, and 23% with ≥ 5 drugs). Therefore, the strategy was changed to conduct individual steps of backward elimination (eliminating all covariates that did not increase the OFV by ≥ 6.635 (*P* ≤ 0.01, with 1 degree of freedom)) prior to the incorporation of a buprenorphine concentration‐related slope, then to use forward stepwise addition to evaluate the concentration‐related slope, the need for diurnal variation, and any other model refinement.

### Evaluation of the effect of BUP‐XR on QT interval

The buprenorphine concentration‐related or norbuprenorphine concentration‐related effects on QTc_Abs_ were tested using a linear model (Eq. (3))

(3) $$\text{QT}=\left[{\text{QTc}}_{\mathrm{Abs}}+C_{\mathrm{bup}}*{\text{SLP}}_{\mathrm{bup}}\right]*{\left(\frac{\text{RR}}{1,000}\right)}^{\mathrm{alpha}}$$

where SLP_bup_ is the slope describing the buprenorphine concentration (C_bup_) (or norbuprenorphine concentration)‐related effects on QTc. IIV was added to the SLP_bup_ assuming a normal distribution.

Similarities in the buprenorphine concentration and norbuprenorphine concentration‐time profile suggest that concentration‐related effects may be indistinguishable. Therefore, buprenorphine concentration and norbuprenorphine concentration‐related slopes were evaluated independently, and the one with the most conservative outcome (i.e., the most likely to have a positive slope) was chosen.

The effect of diurnal variation on QTc_Abs_ was evaluated using a cosine function, but the amplitude was very small (< 1 ms) and diurnal variation was not retained in the model.

### Model performance

A nonparametric bootstrap of the final model was conducted, where the original data were resampled (with replacement) 1,000 times stratified by study. The model was fit to each bootstrapped dataset, and the median values and the 5th and 95th percentiles of the estimated parameters were compared with the estimated parameters from the final model using the original dataset.

A VPC was performed for QT vs. time and plasma concentration vs. QT on study RB‐US‐13‐0001 data, where simulations (n = 1,000) were conducted using the final model with its parameter estimates. A graphical comparison was made between the observed data and the model‐predicted 5th, 50th, and 95th percentiles over time.

### Prediction of drug‐related effects on QTc at therapeutic and supratherapeutic concentrations

The buprenorphine concentration‐related effects on QTc interval were described at therapeutic and supratherapeutic concentrations using a bias‐corrected nonparametric bootstrap procedure to derive two‐sided 90% CIs of the mean predicted effect on QTc.^17^ For therapeutic concentrations, the geometric mean C_max_ obtained at steady state for BUP‐XR 300‐mg or 100‐mg maintenance doses was considered. Supratherapeutic concentrations were those from BUP‐XR 300 mg multiplied by 2.

The following steps were performed:

1. From each bootstrap, the slope was multiplied by the corresponding geometric mean C_max_
2. The two‐sided 90% CI of the concentration‐related change in QTc (ΔQTc) was determined from the distribution across bootstrapped samples
3. A bias‐corrected 90% CI of the mean ΔQTc was determined using the boot package in R, where an additional nonparametric bootstrap was performed

If the upper limit of the 90% CI was < 10 ms, then an effect of the BUP‐XR on QTc was ruled out.

## Funding

The study was sponsored by Indivior Inc.

## Conflict of Interest/Disclosure

V.S., L.C., and L.L. are consultants for Indivior Inc. C.L., A.A., and M.Y. are employees of Indivior Inc.

## Author Contributions

V.S., L.C., L.L., C.L., A.A., and M.Y. wrote the manuscript. V.S. designed the research. V.S., L.L., C.L., A.A., and M.Y. performed the research. V.S. and L.C. analyzed the data.

## Supporting information


**Figure S1.** Goodness‐of‐fit plots including population predictions (PRED) vs. observations (QT), individual predictions (IPRED) vs. QT (B), individual weighted residuals (IWRES) vs. IPRED, and conditional weighted residuals (CWRES) vs. time since first dose for the final concentration‐QT model (model #95).
**Figure S2.** Visual predictive check: QTc over time by sex (0 = males; 1 = females).
**Figure S3.** Visual predictive check: QTc by concentration.
**Figure S4.** Relationship between age and QTcF intervals.

**Table S1.** Summary of concomitant medications.
**Table S2.** Results from sensitivity analyses evaluating the effect of adding IIV onto alpha, using the phase III data alone, and eliminating the effect of age on QTc_Abs._

## References

[1] Wedam, E.F. *et al* QT‐interval effects of methadone, levomethadyl, and buprenorphine in a randomized trial. Arch. Intern. Med. 167, 2469–2475 (2007). 18071169 10.1001/archinte.167.22.2469

[2] Anchersen, K. *et al* Prevalence and clinical relevance of corrected QT interval prolongation during methadone and buprenorphine treatment: a mortality assessment study. Addiction 104, 993–999 (2009). 19392907 10.1111/j.1360-0443.2009.02549.x

[3] Stallvik, M. *et al* Corrected QT interval during treatment with methadone and buprenorphine–relation to doses and serum concentrations. Drug Alcohol Depend. 129, 88–93 (2013). 23084592 10.1016/j.drugalcdep.2012.09.016

[4] Baker, J.R. *et al* Effect of buprenorphine and antiretroviral agents on the QT interval in opioid‐dependent patients. Ann. Pharmacother. 40, 392–396 (2006). 16507617 10.1345/aph.1G524

[5] US Food and Drug Administration (FDA) . Clinical pharmacology and biopharmaceutics review – BuTrans. Food and Drug Administration (2010).

[6] Garnett, C.E. *et al* Methodologies to characterize the QT/– QT interval in the presence of drug‐induced heart rate changes or other autonomic effects. Am. Heart J. 163, 912–930 (2012). 22709743 10.1016/j.ahj.2012.02.023

[7] Haight, B.R. *et al*, Efficacy and safety of a monthly buprenorphine depot injection for opioid use disorder: a multicentre, randomised, double‐blind, placebo‐controlled trial. Lancet 393, 778–790 (2019). 30792007 10.1016/S0140-6736(18)32259-1

[8] Sublocade^TM^ [package insert]. Indivior, North Chesterfield, VA, 2017.

[9] International Conference on Harmonisation of Technical Requirements for Registration of Pharmaceuticals for Human Use. Implementation Working Group ICH E14 Guideline . The clinical evaluation of QT/QTc interval prolongation and proarrhythmic potential for non‐antiarrhythmic drugs. Q&A R3. International Conference on Harmonisation of Technical Requirements for Registration of Pharmaceuticals for Human Use http://www.ich.org/fileadmin/Public_Web_Site/ICH_Products/Guidelines/Efficacy/E14/E14_Q_As_R3__Step4.pdf (2015).

[10] International Conference on Harmonisation of Technical Requirements for Registration of Pharmaceuticals for Human Use . The clinical evaluation of QT/QTc interal prolongation and proarrhythmic potential for non‐antiarrhythmic drugs. International Conference on Harmonisation of Technical Requirements for Registration of Pharmaceuticals for Human Use. https://www.ich.org/fileadmin/Public_Web_Site/ICH_Products/Guidelines/Efficacy/E14/E14_Guideline.pdf (2005).

[11] Fazio, G. *et al* Drugs to be avoided in patients with long QT syndrome: focus on the anaesthesiological management. World J. Cardiol. 5, 87–93 (2013). 23675554 10.4330/wjc.v5.i4.87 PMC3653016

[12] Staikou, C. , Stamelos, M. & Stavroulakis, E. Impact of anaesthetic drugs and adjuvants on ECG markers of torsadogenicity. Br. J. Anaesth. 112, 217–230 (2014). 24305646 10.1093/bja/aet412

[13] Devlin, R.J. & Henry, J.A. Clinical review: major consequences of illicit drug consumption. Crit. Care 12, 202 (2008). 18279535 10.1186/cc6166 PMC2374627

[14] Bey, T. & Patel, A. Phencyclidine intoxication and adverse effects: a clinical and pharmacological review of an illicit drug. Cal. J. Emerg. Med. 8, 9–14 (2007). 20440387 PMC2859735

[15] Darpo, B. *et al* Differentiating the effect of an opioid agonist on cardiac repolarization from micro‐receptor‐mediated, indirect effects on the QT interval: a randomized, 3‐way crossover study in healthy subjects. Clin. Ther. 38, 315–326 (2016). 26749217 10.1016/j.clinthera.2015.12.004

[16] Kienbaum, P. *et al* Chronic mu‐opioid receptor stimulation alters cardiovascular regulation in humans: differential effects on muscle sympathetic and heart rate responses to arterial hypotension. J. Cardiovasc. Pharmacol. 40, 363–369 (2002). 12198322 10.1097/00005344-200209000-00005

[17] Darpo, B. *et al* Results from the IQ‐CSRC prospective study support replacement of the thorough QT study by QT assessment in the early clinical phase. Clin. Pharmacol. Ther. 97, 326–335 (2015). 25670536 10.1002/cpt.60

