# Supplementary material for: Evaluation of the Effects of a Monthly Buprenorphine Depot Subcutaneous Injection on QT Interval During Treatment for Opioid Use Disorder
Source: Clin Pharmacol Ther. 2019 Apr 8;106(3):576–84. doi: 10.1002/cpt.1406 (PMC6766787; doi:10.1002/cpt.1406)
Supplement: Supplementary file 1 — Figure S1. Goodness‐of‐fit plots including population predictions (PRED) vs. observations (QT), individual predictions (IPRED) vs. QT (B), individual weighted residuals (IWRES) vs. IPRED, and conditional weighted residuals (CWRES) vs. time since first dose for the final concentration‐QT model (model #95). Figure S2. Visual predictive check: QTc over time by sex (0 = males; 1 = females). Figure S3. Visual predictive check: QTc by concentration. Figure S4. Relationship between age and QTcF intervals. [file CPT-106-576-s001.pdf]

Figure S1. Goodness of Fit Plots Including Predicted (PRED) vs Observed (QT), Individual Predicted (IPRED) vs QT (B), Individual Weighted Residuals (IWRES) vs IPRED, and Conditional Weighted Residuals (CWRES) vs Time Since First Dose for the Final Concentration-QT Model (Model #95)

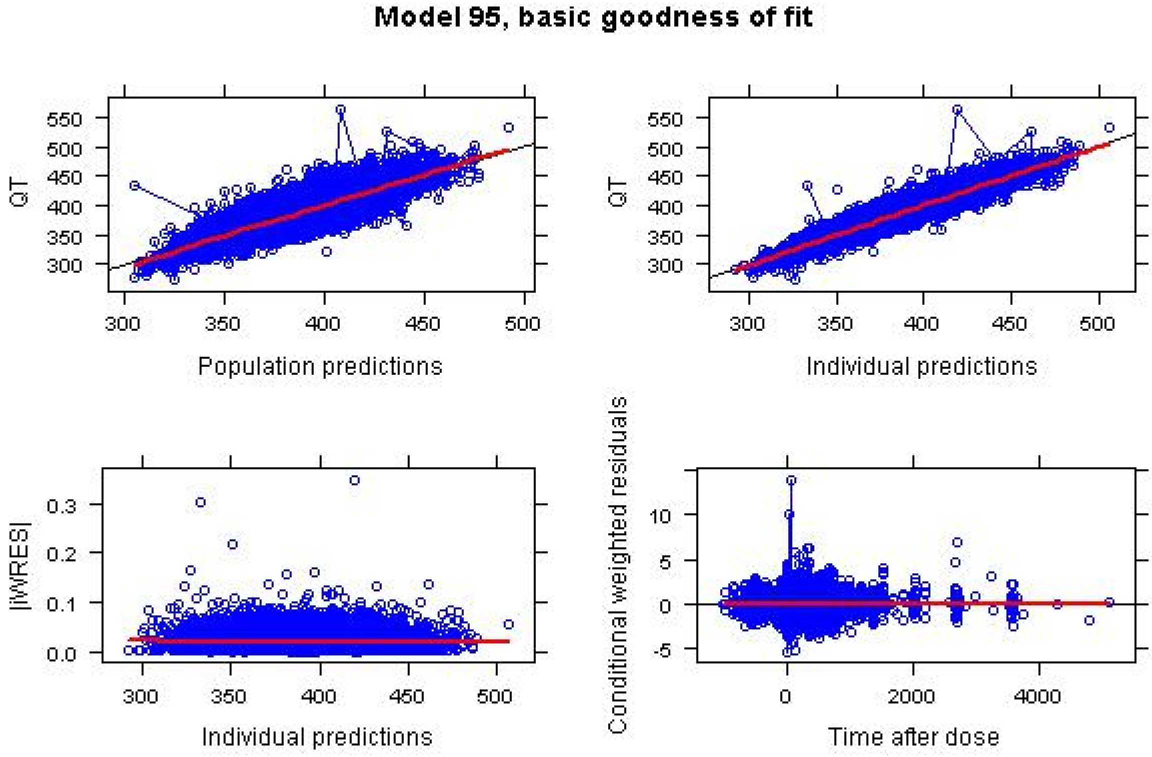

Figure S2: Visual Predictive Check: QTc Over Time by Sex (0=males; 1=females)

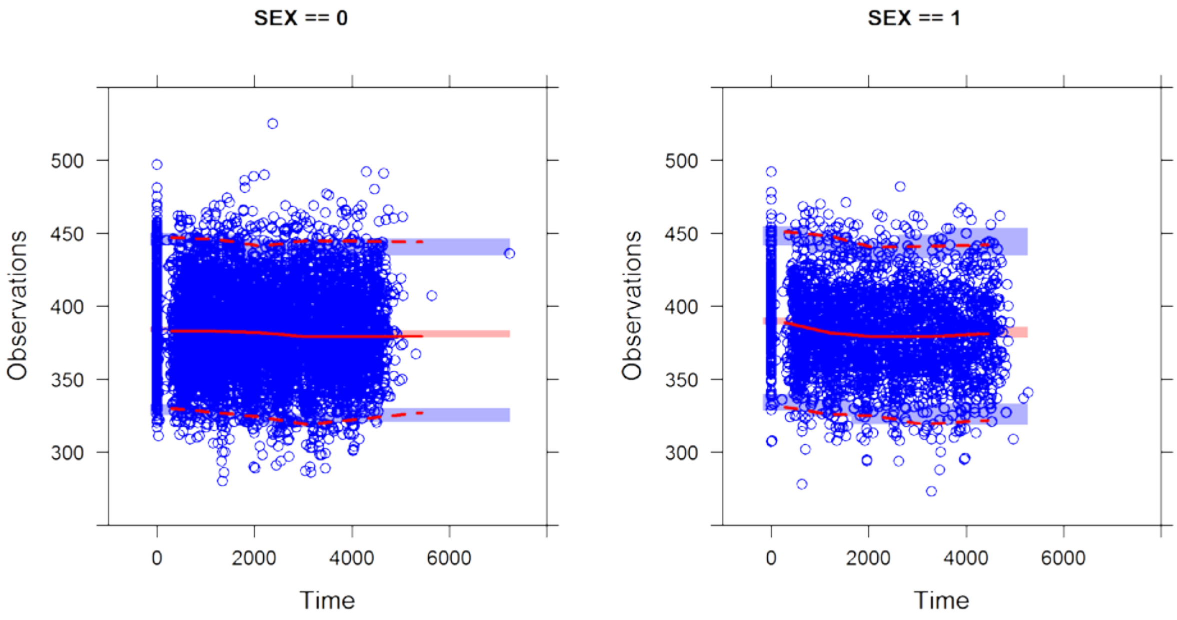

Figure S3: Visual Predictive Check: QTc by Concentration

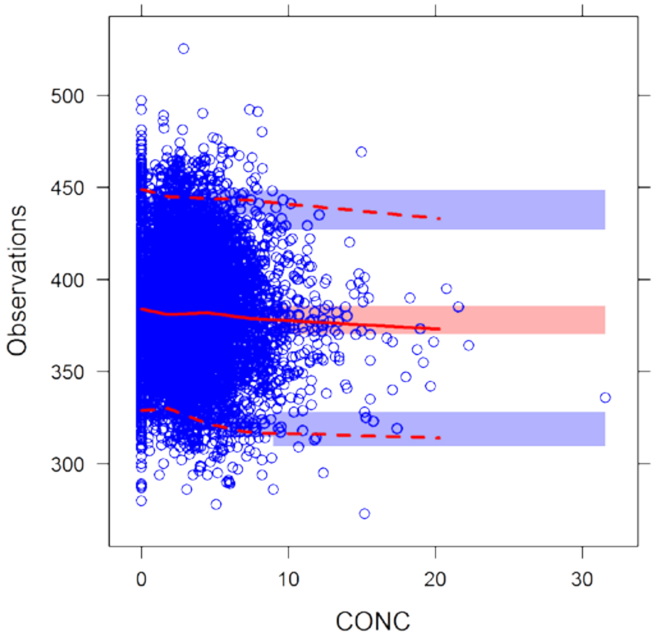

Figure S4: Relationship between Age and QTcF Intervals

Reduced Dataset	Full Dataset
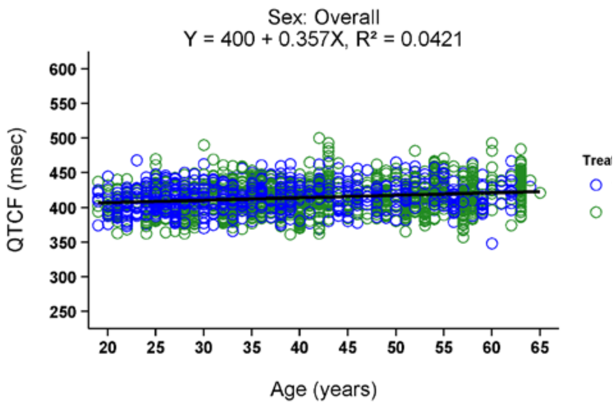	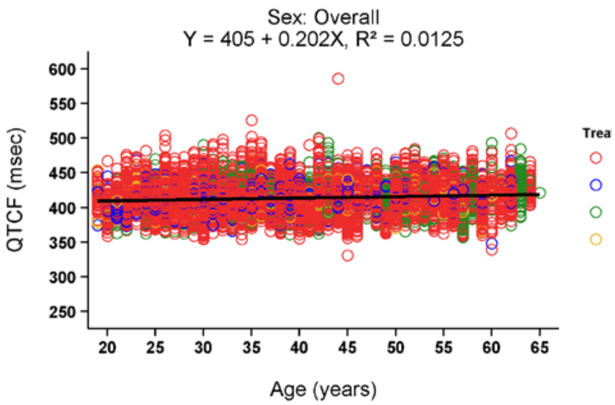
