# Supplementary material for: Evaluation of the Effects of a Monthly Buprenorphine Depot Subcutaneous Injection on QT Interval During Treatment for Opioid Use Disorder
Source: Clin Pharmacol Ther. 2019 Apr 8;106(3):576–84. doi: 10.1002/cpt.1406 (PMC6766787; doi:10.1002/cpt.1406)
Supplement: Supplementary file 2 — Table S1. Summary of concomitant medications. Table S2. Results from sensitivity analyses evaluating the effect of adding IIV onto alpha, using the phase III data alone, and eliminating the effect of age on QTcAbs. [file CPT-106-576-s002.pdf]

Table S1: Summary of Concomitant Medications

Characteristic	Statistic	10-0011	11-0020	12-0005	13-0001	13-0006	Overall
**Methocarbamol: No Use**	**N (%)**	1 (8.33%)	7 (14.6%)	44 (36.1%)	855 (98.7%)	43 (65.2%)	950 (85.3%)
**Methocarbamol: Use**	**N (%)**	11 (91.7%)	41 (85.4%)	78 (63.9%)	11 (1.27%)	23 (34.8%)	164 (14.7%)
**Clonidine: No Use**	**N (%)**	0 (0%)	7 (14.6%)	52 (42.6%)	842 (97.2%)	66 (100%)	967 (86.8%)
**Clonidine: Use**	**N (%)**	12 (100%)	41 (85.4%)	70 (57.4%)	24 (2.77%)	0 (0%)	147 (13.2%)
**Hydroxyzine: No Use**	**N (%)**	0 (0%)	3 (6.25%)	35 (28.7%)	811 (93.6%)	32 (48.5%)	881 (79.1%)
**Hydroxyzine: Use**	**N (%)**	12 (100%)	45 (93.8%)	87 (71.3%)	55 (6.35%)	34 (51.5%)	233 (20.9%)
**Oxycodone: No Use**	**N (%)**	5 (41.7%)	14 (29.2%)	68 (55.7%)	706 (81.5%)	49 (74.2%)	842 (75.6%)
**Oxycodone: Use**	**N (%)**	7 (58.3%)	34 (70.8%)	54 (44.3%)	160 (18.5%)	17 (25.8%)	272 (24.4%)
**Cocaine: No Use**	**N (%)**	10 (83.3%)	31 (64.6%)	95 (77.9%)	484 (55.9%)	39 (59.1%)	659 (59.2%)
**Cocaine: Use**	**N (%)**	2 (16.7%)	17 (35.4%)	27 (22.1%)	382 (44.1%)	27 (40.9%)	455 (40.8%)
**Amphetamine: No Use**	**N (%)**	12 (100%)	31 (64.6%)	101 (82.8%)	671 (77.5%)	43 (65.2%)	858 (77.0%)
**Amphetamine: Use**	**N (%)**	0 (0%)	17 (35.4%)	21 (17.2%)	195 (22.5%)	23 (34.8%)	256 (23.0%)
**Methadone: No Use**	**N (%)**	4 (33.3%)	19 (39.6%)	117 (95.9%)	757 (87.4%)	64 (97.0%)	961 (86.3%)
**Methadone: Use**	**N (%)**	8 (66.7%)	29 (60.4%)	5 (4.10%)	109 (12.6%)	2 (3.03%)	153 (13.7%)
**Cannabinoid: No Use**	**N (%)**	10 (83.3%)	14 (29.2%)	48 (39.3%)	511 (59.0%)	30 (45.5%)	613 (55.0%)
**Cannabinoid: Use**	**N (%)**	2 (16.7%)	34 (70.8%)	74 (60.7%)	355 (41.0%)	36 (54.5%)	501 (45.0%)
**Barbiturate: No Use**	**N (%)**	12 (100%)	45 (93.8%)	122 (100%)	842 (97.2%)	65 (98.5%)	1086 (97.5%)
**Barbiturate: Use**	**N (%)**	0 (0%)	3 (6.25%)	0 (0%)	24 (2.77%)	1 (1.52%)	28 (2.51%)
**Methamphetamine: No Use**	**N (%)**	4 (33.3%)	20 (41.7%)	110 (90.2%)	781 (90.2%)	58 (87.9%)	973 (87.3%)
**Methamphetamine: Use**	**N (%)**	8 (66.7%)	28 (58.3%)	12 (9.84%)	85 (9.82%)	8 (12.1%)	141 (12.7%)
**Phencyclidine: No Use**	**N (%)**	12 (100%)	46 (95.8%)	114 (93.4%)	832 (96.1%)	66 (100%)	1070 (96.1%)
**Phencyclidine: Use**	**N (%)**	0 (0%)	2 (4.17%)	8 (6.56%)	34 (3.93%)	0 (0%)	44 (3.95%)
**Codeine: No Use**	**N (%)**	12 (100%)	47 (97.9%)	120 (98.4%)	351 (40.5%)	66 (100%)	596 (53.5%)
**Codeine: Use**	**N (%)**	0 (0%)	1 (2.08%)	2 (1.64%)	515 (59.5%)	0 (0%)	518 (46.5%)
**Hydrocodone: No Use**	**N (%)**	12 (100%)	25 (52.1%)	104 (85.2%)	735 (84.9%)	65 (98.5%)	941 (84.5%)
**Hydrocodone: Use**	**N (%)**	0 (0%)	23 (47.9%)	18 (14.8%)	131 (15.1%)	1 (1.52%)	173 (15.5%)
**Hydromorphone: No Use**	**N (%)**	0 (0%)	42 (87.5%)	117 (95.9%)	327 (37.8%)	66 (100%)	552 (49.6%)
**Hydromorphone: Use**	**N (%)**	12 (100%)	6 (12.5%)	5 (4.10%)	539 (62.2%)	0 (0%)	562 (50.4%)
**Morphine: No Use**	**N (%)**	12 (100%)	42 (87.5%)	116 (95.1%)	279 (32.2%)	66 (100%)	515 (46.2%)
**Morphine: Use**	**N (%)**	0 (0%)	6 (12.5%)	6 (4.92%)	587 (67.8%)	0 (0%)	599 (53.8%)
**Oxymorphone: No Use**	**N (%)**	12 (100%)	47 (97.9%)	120 (98.4%)	721 (83.3%)	66 (100%)	966 (86.7%)
**Oxymorphone: Use**	**N (%)**	0 (0%)	1 (2.08%)	2 (1.64%)	145 (16.7%)	0 (0%)	148 (13.3%)
**Heroin: No Use**	**N (%)**	12 (100%)	26 (54.2%)	33 (27.0%)	866 (100%)	66 (100%)	1003 (90.0%)
**Heroin: Use**	**N (%)**	0 (0%)	22 (45.8%)	89 (73.0%)	0 (0%)	0 (0%)	111 (9.96%)

Use = subject received the concomitant medication at any point during the study, No Use = subject did not receive the concomitant medication at any point during the study

Table S2: Results from Sensitivity Analysis Evaluating the Effect of Adding IIV onto Alpha, Using the Phase 3 Data Alone, and Eliminating the Effect of Age on QTcAbs

	Final Model		Adding IIV on Alpha		Phase 3 data Only		Eliminating Age	
	**Estimate**	**%RSE**	**Estimate**	**%RSE**	**Estimate**	**%RSE**	**Estimate**	**%RSE**
QTcAbs (msec)	400	0.2	400	0.229	400	0.1	411	0.2
Alpha	0.333		0.333		0.333		0.333	
Sex on QTcAbs	0.0189	13.7	0.0196	13.7	0.018	17.1	0.0164	16.3
COWS on alpha	0.00151	52.1	0.00157	43.2	0.00312	24.8	0.0016	49.5
Hydroxyzine on QTcAbs	0.00423	35.7	0.00473	31.3	0.0079	34.1	0.00398	37.4
Methadone on QTcAbs	0.0153	18.4	0.0156	18.2	0.00293	76.1	0.0148	18.4
Age on QTcAbs	0.324	7	0.326	7.2	0.334	4.1		
Holter vs non-Holter on QTcAbs	-0.00423	24.6	-0.00314	31.5	-0.00426	24.4	-0.00409	24.7
Central vs non-central reading on QTcAbs	-0.021	11.5	-0.0195	13.5	-0.021		-0.0166	16.3
Concentration-related slope (msec/ng/mL)	-0.0507	168.8	-0.0604	131	-0.0157	595.5	-0.0431	200.9
Codeine on QTcAbs	0.00327	35.2	0.00318	34.3	0.00382	30.1	0.00326	34.4
Oxycodone on QTcAbs	-0.00378	43.4	-0.00332	47.0	-0.00205	104.9	-0.00392	40.8
Phencyclidine on QTcAbs	0.00861	44.1	0.00958	40.6	0.0109	40.9	0.00844	43.6
Barbiturates on QTcAbs	0.0124	38.9	0.0116	42.2	0.0135	35	0.0119	39.3
Cocaine on QTcAbs	0.00428	28	0.00429	28.4	0.00322	40.1	0.00438	26.7
IIV on QTcAbs (±msec)	14.3	5.2	14.4	5.3	14.8	6.1	14.7	5.2
IIV on concentration-related slope (±msec/ng/mL)	0.767	33.1	0.581	38.9	0.7714	30.1	0.7609	35.8
IIV on alpha	NA		0.0535	14.7	NA		NA	
Residual error (±msec)	10.6	3.3	10.2	3.17	10.3	2.6	10.6	3.3
